# Improving the overall survival prognosis prediction accuracy: A 9‐gene signature in CRC patients

**DOI:** 10.1002/cam4.4104

**Published:** 2021-08-04

**Authors:** Wenbo Zheng, Yijia Lu, Xiaochuang Feng, Chunzhao Yang, Ling Qiu, Haijun Deng, Qi Xue, Kai Sun

**Affiliations:** ^1^ Department of General Surgery & Guangdong Province Key Laboratory of Precision Medicine for Gastrointestinal Tumor The First School of Clinical Medicine Nanfang Hospital Southern Medical University Guangzhou China; ^2^ Department of Obstetrics and Gynaecology Nanfang Hospital Southern Medical University Guangzhou China; ^3^ Department of General Surgery Traditional Chinese and Western Medicine Hospital Southern Medical University Guangzhou China

**Keywords:** CRC, nomogram, prognosis, survival‐related genes, WGCNA

## Abstract

Colorectal cancer (CRC) is a malignant tumor and morbidity rates are among the highest in the world. The variation in CRC patients' prognosis prompts an urgent need for new molecular biomarkers to improve the accuracy for predicting the CRC patients' prognosis or as a complement to the traditional TNM staging for clinical practice. CRC patients' gene expression data of HTSeq‐FPKM and matching clinical information were downloaded from The Cancer Genome Atlas (TCGA) datasets. Patients were randomly divided into a training dataset and a test dataset. By univariate and multivariate Cox regression survival analyses and Lasso regression analysis, a prediction model which divided each patient into high‐or low‐risk group was constructed. The differences in survival time between the two groups were compared by the Kaplan–Meier method and the log‐rank test. The weighted gene co‐expression network analysis (WGCNA) was used to explore the relationship between all the survival‐related genes. The survival outcomes of patients whose overall survival (OS) time were significantly lower in the high‐risk group than that in the low‐risk group both in the training and test datasets. Areas under the ROC curves which termed AUC values of our 9‐gene signature achieved 0.823 in the training dataset and 0.806 in the test dataset. A nomogram was constructed for clinical practice when we combined the 9‐gene signature with TNM stage and age to evaluate the survival time of patients with CRC, and the C‐index increased from 0.739 to 0.794. In conclusion, we identified nine novel biomarkers that not only are independent prognostic indexes for CRC patients but also can serve as a good supplement to traditional clinicopathological factors to more accurately evaluate the survival of CRC patients.

## Graphical abstract

We identified a 9‐gene signature screened out based on batch survival analysis that can be used not only as an independent prognostic index for CRC patients but also as a favorable supplement to traditional clinicopathological factors for clinicians to more accurately and effectively evaluate the survival of CRC patients.

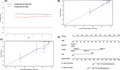

## 1 INTRODUCTION

Colorectal cancer (CRC) tops the list of high‐risk cancers that endangered public life over the past several decades, especially in the western developed countries.^1^, ^2^ It is estimated that as of January 1, 2019, more than 1.5 million men and women have been diagnosed with CRC in the United States and 145,600 new cases will be diagnosed in 2019.^3^ Therefore, a novelty intervention or blocking‐up strategy of this disease appears to be particularly important against the CRC for patients to live a longer or a high‐quality life. The survival time of patients with CRC varies greatly in different stages. For example, the 5‐year survival rate of patients with stage I and II patients is 91 and 82%, respectively, while those of stage IV CRC are approximately 12% [3]. Over the past several decades, the mortality rates of CRC patients have reduced moderately due to early diagnosis and treatment.^3^, ^4^, ^5^ However, there is no effective quantitative prognostic index for CRC patients. In current clinical practice, clinicians judge the prognosis of patients with CRC mainly based on the tumor–node–metastasis (TNM) staging and clinical characteristics of the disease.^6^ Vague judgment criteria not only aggravate the psychological burden of patients but also increase the confusion of clinicians to a certain extent. Therefore, there is an urgent need for an accurate and highly feasible prediction tool in which clinicians can serve patients more confidently.

To our knowledge, several researchers have proposed a variety of strategies that can better predict the survival of patients with CRC. For example, Bingrong et al identified an effective 6‐lncRNA signature with good performance that divides CRC patients into high‐and low‐risk groups that have significantly different survival times.^7^ However, they have not further proven its clinical application and the highest area under the curve (AUC) value of their signature was 0.733, indicating that the stability and reliability of the model are at an average level. Zheng Zhou et al identified a 5‐autophagy‐related gene signature that can divide CRC patients into low‐and high‐risk groups and developed a nomograph to guide individualized treatment.^8^ Unfortunately, the AUC value only reached 0.58 when they tried to demonstrate the performance of their formula in other datasets, indicating that the reliability of the model is not optimal. We observed that the same score in the nomograph chart corresponds to the same 1‐year and 5‐year survival rates, which are unreasonable. Therefore, the purpose of our research emerged and we aimed to construct a more stable and effective signature that can effectively predict the OS of CRC patients to give patients a more accurate answer in clinical practice.

In the present study, by a large CRC cohort from The TCGA database, we developed a credible expression‐based 9‐gene signature that divides patients into high‐and low‐risk groups with significant differences in OS. In the validation process, the signature in our study also reflected high reliability in the test dataset. Combined with clinicopathological factors such as the TNM stage, which has been used in the clinic, the signature can provide a more accurate prediction for individual patients during clinical work.

## 2 MATERIALS AND METHODS

### 2.1 Data sources and research design

We downloaded the HTSeq‐FPKM gene expression data and corresponding clinical information of all postoperative CRC patients prior to October 2019 from the official website of the TCGA database (https://www.cancer.gov). A total of 530 samples was obtained in the aggregate dataset, including 42 normal tissue samples and 488 CRC tissue samples. After the exclusion of patients with incomplete survival information, a total of 467 patients with complete follow‐up data was included. A GEO dataset GSE103479 downloaded from the official website (https://www.ncbi.nlm.nih.gov/geo/) including 156 postoperative drug‐resistant stage II and stage III CRC samples was used during the further validating process (Table 1). In this study, initially, genes with an average expression level lower than 0.1 in all samples were excluded and batch survival analysis by which 1464 genes discovered to be statistically significant (*p* value < 0.05) was conducted to screen the genes associated with patients’ OS using the “survival” package in R (version 3.6.0). Univariate Cox regression analysis was used to further identify genes that were screened by previous batch survival analysis associated with patients' OS and we set the filter criteria as HR>1.5 or HR<0.8 and *p* value < 0.01. Finally, 91 eligible genes were obtained. Then, we used the “caret” package in R to randomly divide 467 samples into two groups, in which 235 samples were allocated to the training group and 232 samples were allocated to the test group.

**TABLE 1:** Information of CRC patients in the training dataset and test dataset from TCGA database and validation dataset from the GEO database

Variables	TCGA training dataset	TCGA test dataset	GEO validation dataset
Age (mean, range)	66.8 (31‐90)	66.3 (33‐90)	69.7 (36.6‐94)
**Gender**			
Male	130	111	87
Female	105	121	67
**TMN stage**			
Stage I	43	39	
Stage II	92	85	83
Stage III	56	69	71
Stage IV	39	30	
Unknown	5	9	
T stage			
Tis	0	1	
T1	5	9	1
T2	44	39	6
T3	162	156	109
T4	24	27	38
M stage			
M0	172	175	86
M1	38	30	
Mx	20	25	68
Unknown	5	2	
N stage			
N0	141	134	83
N1	57	55	50
N2	36	43	21
Nx	1	0	
Tumor location			
Colon	196	183	127
Rectum	39	49	27
Total	235	232	154

Abbreviations: CRC, colorectal cancer; GEO, Gene Expression Omnibus; TCGA, The Cancer Genome Atlas.

**FIGURE 1:**
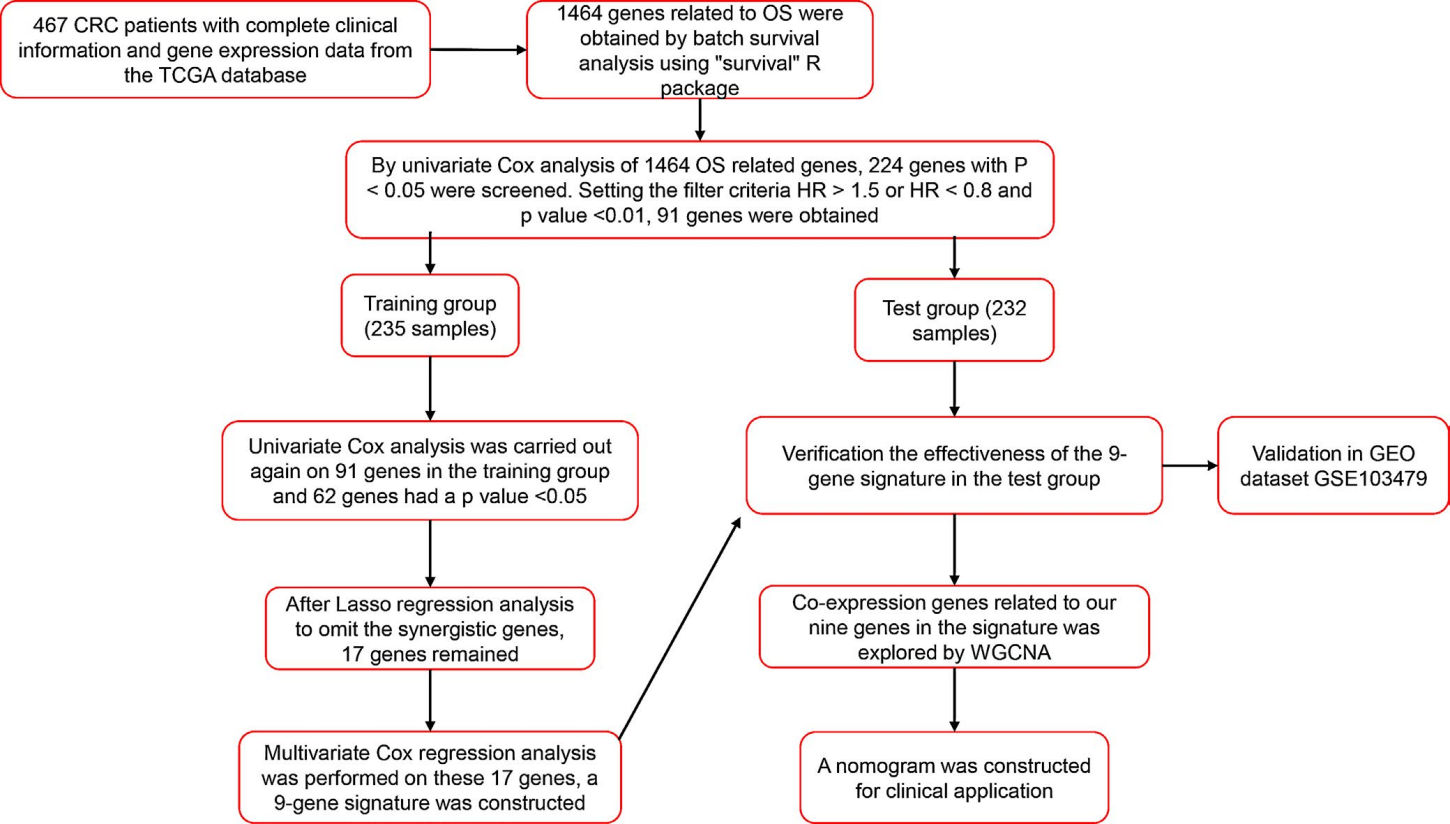
The workflow of the identification of CRC OS‐related 9‐gene signature

In the training group, 91 genes were rescreened using univariate Cox regression analysis and 62 genes with *p* value < 0.05 were obtained. Then, we continued to perform Lasso regression analysis of these 62 genes in the training group to omit the synergistic genes and obtained 17 genes. Finally, by using multi‐cox regression analysis on these 17 genes in the training group, a 9 genes prognosis prediction model was established to predict the OS rate of CRC patients. The stability and effectiveness of the model were verified in the test group and validation dataset. Details in this research are shown in Figure 1.

### 2.2 Construction of prognostic signature

A multivariate Cox proportional hazards regression model was established and used to evaluate the pertinence between the expression value of the 9‐gene signature and CRC patients' OS. The risk score of each patient was constructed by weighting the expression value of prognostic genes according to their multivariate Cox regression coefficients. The formula is shown below:

$$\text{Risk Score}\;\left(\text{RS}\right)=\sum_{i=1}^{n}C_{i}\cdot Exp_{i}$$

where *n* is the number of prognostic genes, *Exp* ***_i_*** is the expression value of gene *i*, and C***_i_*** is the regression coefficient of gene *i* in the multivariate Cox regression analysis. The risk scores of each patient were distributed into two groups, high and low‐risk groups, by setting the median value as the demarcation point. The “survival” R package was used to distinguish the survival differences between the high‐and low‐risk groups. The time‐dependent receiver operating characteristic (ROC) analysis based on the “SurvivalROC” R package was used to assess the predictive stability and effectiveness of the 9‐gene signature in each group. To verify whether this 9‐gene signature can serve as an independent prognostic index compared with the existing traditional clinicopathological indicators for evaluating prognosis, we performed a multivariate regression analysis that takes into account our 9‐gene signature, TNM stage, age, sex, and so on. Finally, a nomogram and related calibration curves were established based on CRC patients for further clinical application.

### 2.3 WGCNA

To explore the relationship between the nine genes in our signature and all the survival‐related genes mentioned above, the weighted gene co‐expression network analysis (WGCNA) without clinical traits was analyzed. By selecting 5 as the soft threshold, a weighted gene co‐expression network was constructed by the R “WGCNA” package with approximate scale‐free properties. A highly synergistic matrix of genes was determined by the relevance among the expression values of all those genes. The network module was produced by the topological overlap measurement (TOM)^9^ and using the dynamic hybrid cutting method (a bottom‐up algorithm) to identify the co‐expression gene modules.^10^ Eventually, the modules with related genes were merged. The correlation between genes and modules was measured by calculating gene significance (GS) and module significance (MS). The target genes in the related modules were visualized by Cytoscape 3.6.1 software.

### 2.4 Statistical analysis

The survival‐related genes and survival differences between the high‐and low‐risk groups in the training dataset and test dataset were obtained by the “survival” R package through the Kaplan–Meier method and compared by the log‐rank test. Lasso regression analysis was performed by the “glmnet” R package to eliminate synergistic genes. The random grouping was completed by the "caret" R package. Time‐dependent ROC curve was used to describe the sensitivity and specificity of survival prediction based on the risk score generated by the “survivalROC” R package. Multivariate Cox regression analysis was used to show the independent predictability of the signature and generate a nomogram. The calibration curve was used to evaluate whether patients' actual survival was consistent with that predicted by the nomogram. The nomogram and calibration curve were generated by the “rms” R package. The “pec” R package was used to compare the concordance index between our nomogram and nowadays TNM stage added with age through C‐index function. All statistical tests were two‐sided and *p* < 0.05 was regarded as statistically significant. The statistical analysis was performed in R software (version 3.6.0).

## 3 RESULTS

### 3.1 Gene selection and prognostic signature construction

**FIGURE 2:**
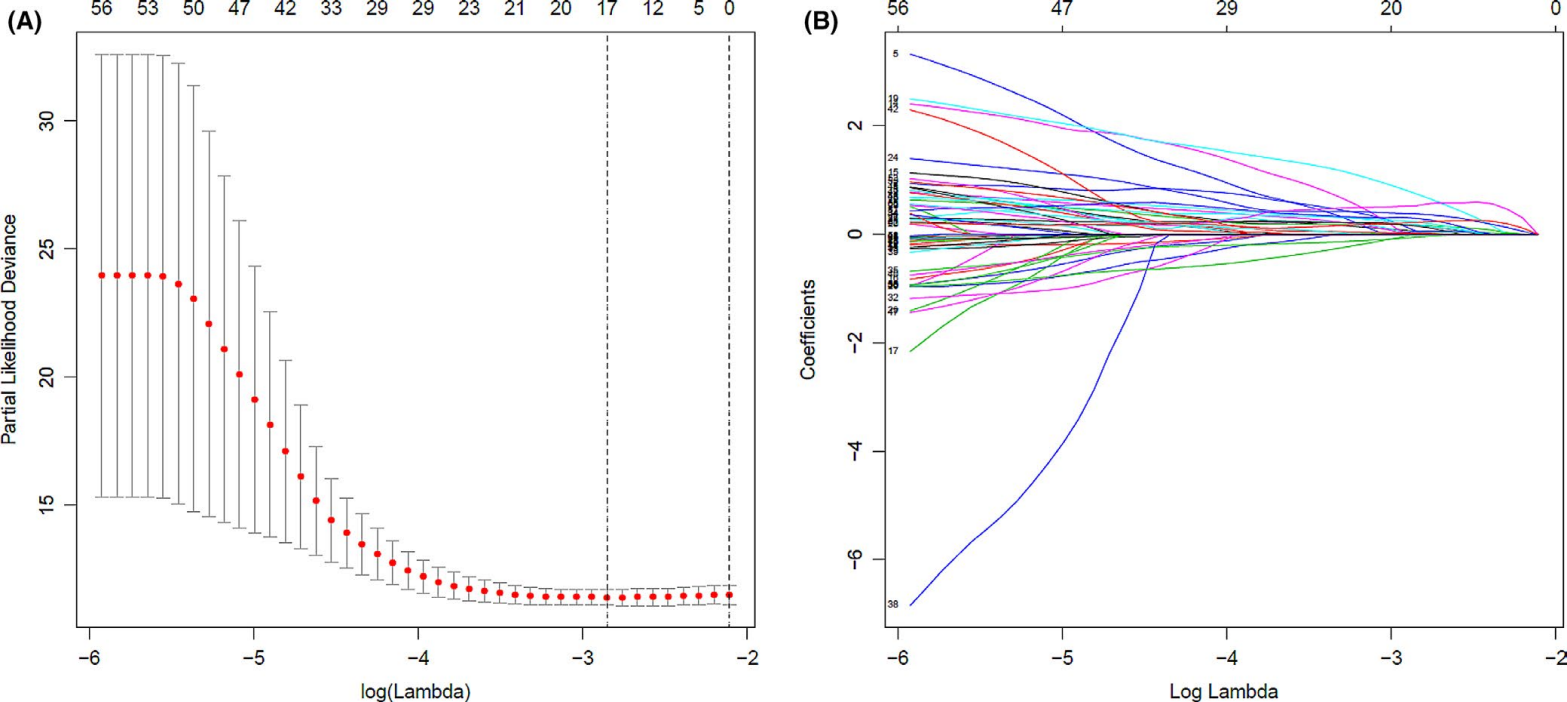
Seventeen genes were screened out by Lasso regression analysis of the 62 genes in the training dataset. (A) The longitudinal solid line represents the partial likelihood deviation ±standard error and the longitudinal dotted line indicates that the best parameter is selected according to the minimum value (left) and 1‐SE (right). Lambda is the tuning parameter. (B) y axis represents Coefficients. Each curve in the graph corresponds to the value of each characteristic regression coefficient varying with the log(Lambda) value

In our study, 1464 genes correlated with OS in patients diagnosed with CRC were obtained through the Kaplan–Meier method. Univariate Cox proportional hazards regression analysis was performed on these 1464 genes and 224 of them were found to be statistically significant with a *p* value lower than 0.05. Setting the filtered standard as HR>1.5 or HR<0.8 and *p* value < 0.01, 91 genes were selected for further research. A total of 467 CRC patients was divided randomly into two groups: a training group (*n* = 235) and a test group (*n* = 232). Univariate Cox regression analysis was used again to verify the effect of the 91 genes on the OS of CRC patients in the training group and 62 genes achieved a statistical significance. To improve the interpretability and prediction accuracy of the regression model and to solve the problem of collinearity of variables, we performed Lasso regression analysis of the 62 genes in the training dataset and the results showed that 17 genes were screened out as the basis for our further construction of a model to predict the OS of CRC patients (Figure 2). Finally, multivariate Cox regression analysis in the training group was carried out on those 17 genes, and a signature that predicted the OS of patients with CRC consisting of 9 genes was established. The overall information of these nine genes is shown in Table 2.

**TABLE 2:** Overall information of the nine prognostic genes in our signature

Ensembl ID	Gene symbol	Location	Coefficient	*p* value	Description
ENSG 00 000 104 892	KLC3	Chr19: 45,333,434‐45,351,520(+)	0.417	0.037	Kinesin light chain 3
ENSG 00 000 205 704	LINC00634	Chr 22: 41,952,174‐41,958,933(+)	1.131	0.029	Long intergenic non‐protein coding RNA 634
ENSG 00 000 257 108	NHLRC4	Chr 16: 567,005‐569,495(+)	2.155	0.0007	NHL repeat containing 4
ENSG 00 000 174 370	C11orf45	Chr 11: 128,899,565‐128,906,069(−)	0.979	0.005	Chromosome 11 open reading frame 45
ENSG 00 000 155 592	ZKSCAN2	Chr 16: 25,236,001‐25,257,845(−)	0.417	0.009	Zinc finger with KRAB and SCAN domains 2
ENSG 00 000 088 727	KIF9	Chr 3: 47,228,026‐47,283,451(−)	−0.462	0.022	Kinesin family member 9
ENSG 00 000 166 813	KIF7	Chr 15: 89,608,789‐89,655,467(−)	0.522	0.002	Kinesin family member 7
ENSG 00 000 103 449	SALL1	Chr 16: 51,135,975‐51,151,367(−)	−0.741	0.119	Spalt‐like transcription factor 1
ENSG 00 000 181 781	ODF3L2	Chr 19: 463,346‐474,983(−)	0.71	0.084	Outer dense fiber of sperm tails 3 like 2

### 3.2 9‐gene prognostic signature verification and comparison

**FIGURE 3:**
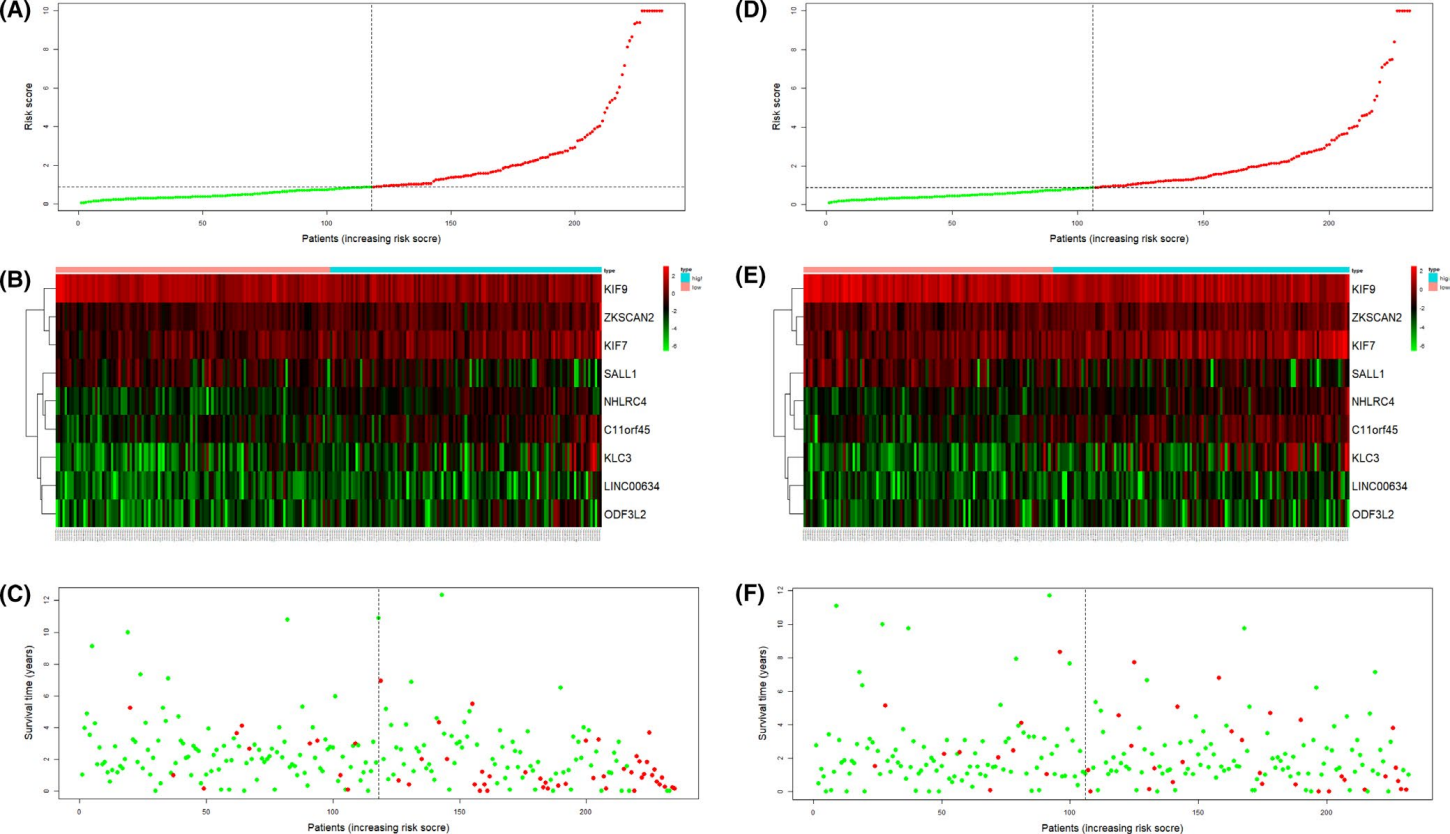
The risk score distribution, gene expression, and CRC patients’ survival status in the training (A–C) and test (D–F) datasets are based on the risk score of the 9‐gene signature

**FIGURE 4:**
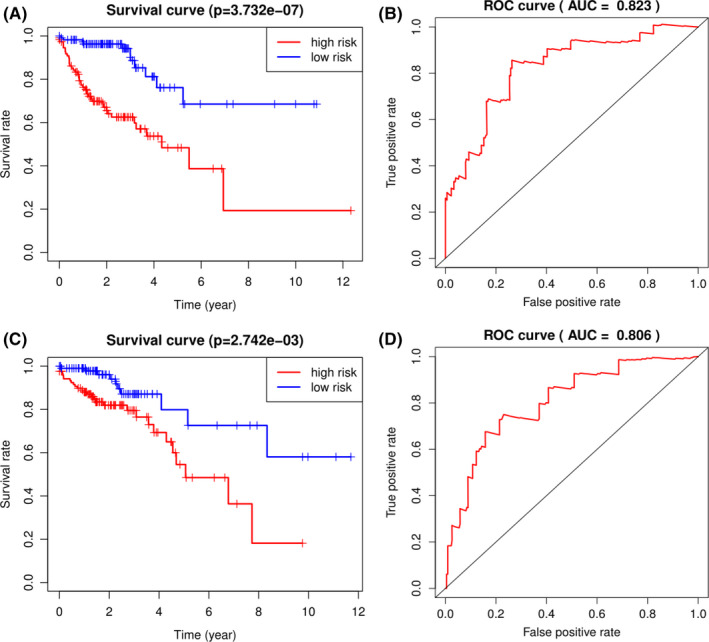
The OS time of patients in the high‐and low‐risk groups in the training (A,B) and test (C,D) datasets and the corresponding ROC curve in which the AUC values reached 0.823 in the training dataset and 0.806 in the test dataset

**FIGURE 5:**
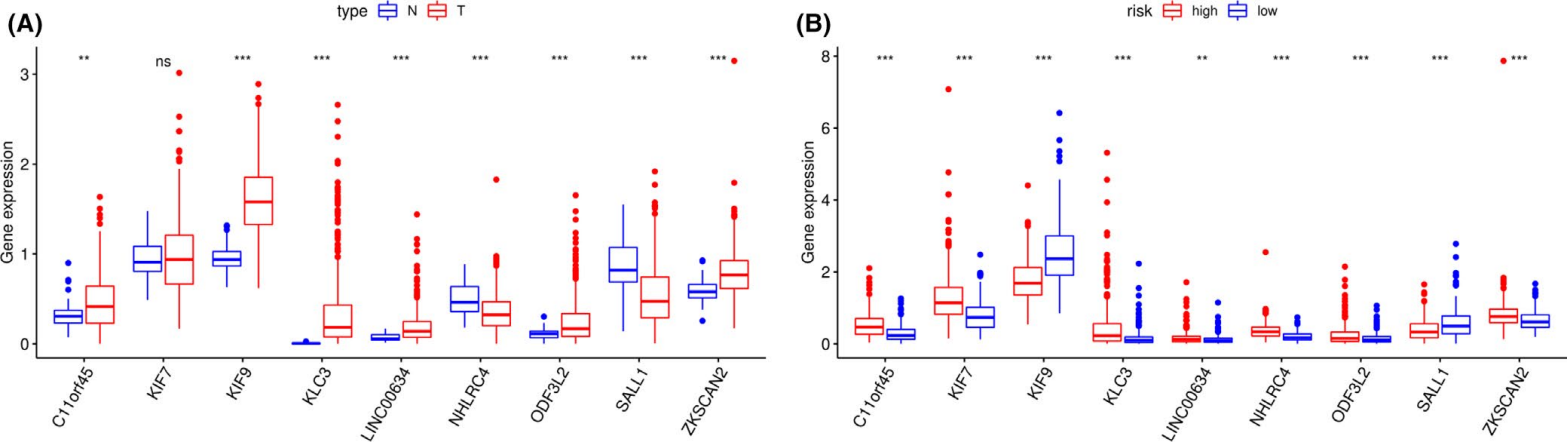
The expression varieties of these nine genes between CRC tumor tissues and normal tissues (A) and between the high‐and low‐risk groups (B)

**FIGURE 6:**
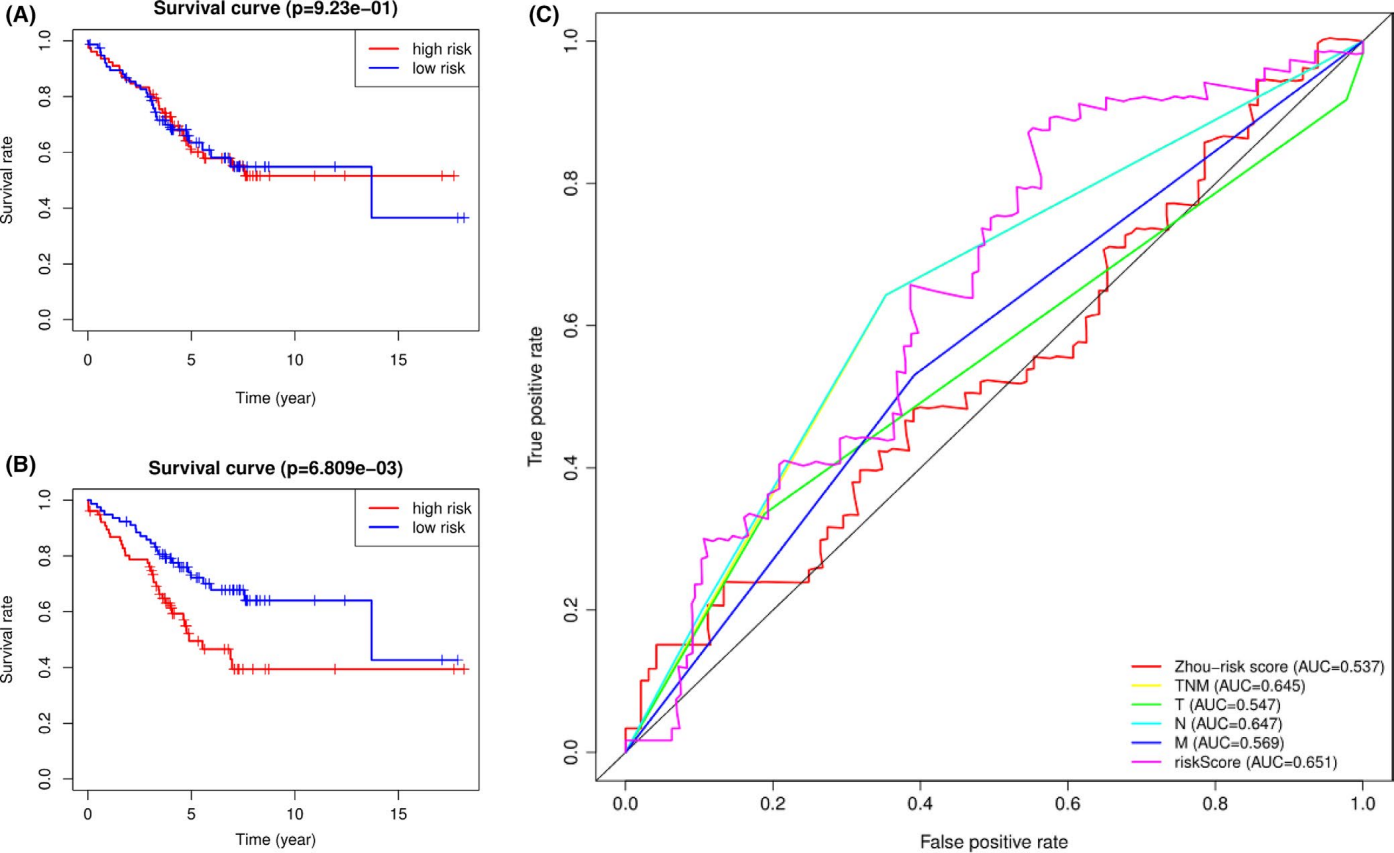
Validation and comparison of the 9‐gene signature with other researcher's prognosis prediction model and TNM stage in GEO dataset. (A) The discrimination of CRC patient's OS time by Zhou's 5‐gene signature. (B) A multi‐ROC curve showed a comparison of AUC values between the 9‐gene signature and other factors including Zhou's signature and TNM stage. (C) The difference in OS time between high‐risk and low‐risk groups based on the 9‐gene signature

Based on multivariate Cox analysis and obtained regression coefficients, a prognostic signature was established, by which patients’ risk score was calculated. Patients in both the training and test datasets were divided into high‐risk and low‐risk groups by setting the median risk score as a demarcation point. The risk score distribution, gene expression, and CRC patients’ survival status of the two risk groups in the training and test datasets are shown in Figure 3. The results in both the training and test datasets showed that the high‐risk groups lives obviously shorter than the low‐risk groups reflected in the OS time. The time‐dependent ROC curve showed that the 9‐gene signature reached AUC values of 0.823 in the training dataset and 0.806 in the test dataset (Figure 4), indicating a substantially effective performance of the OS prediction. Of the nine genes, seven were related to a high risk of CRC (KLC3, LINC00634, NHLRC4, C11orf45, ZKSCAN2, KIF7, and ODF3L2; HR > 1), and two seemed to be protective genes (KIF9 and SALL1; HR < 1). We examined and compared the differential expression of the nine prognostic genes between CRC tumor tissues and normal tissues and between the high‐risk and low‐risk groups. Except for KIF7, the remaining genes were differentially expressed between tumor and normal tissues and were statistically significant. Patients with high‐risk scores are more likely to express risk‐related genes, while patients with low‐risk scores tended to express protective genes. The expression varieties of these genes are shown in Figure 5. To explore the effectiveness of the predicting signature constructed by those nine genes, after performing the Cox proportional hazards regression analyzes, GEO dataset GSE103479 showed a significant difference in the OS time between high and low‐risk groups (Figure 6B). Further exploring work was performed to ensure whether our 9‐gene signature was valuable for more research, we compared the signature with a 5‐autophagy‐related gene model constructed by Zhou mentioned above on distinguishing OS time. Unfortunately, Zhou's signature was unable to distinguish CRC patient's OS time in dataset GSE103479 (Figure 6A). The stability of our 9‐gene signature in predicting CRC patient’ prognosis was also verified by comparing with the current gold standard TNM stage using time‐dependent ROC curve, in which the AUC value on 5 years of our 9‐gene signature achieved 0.651 and TNM stage with a value of 0.645, while Zhou's signature only reached 0.537 (Figure 6C).

### 3.3 The prognostic characteristics of the 9‐gene signature are independent of other clinicopathological factors

**FIGURE 7:**
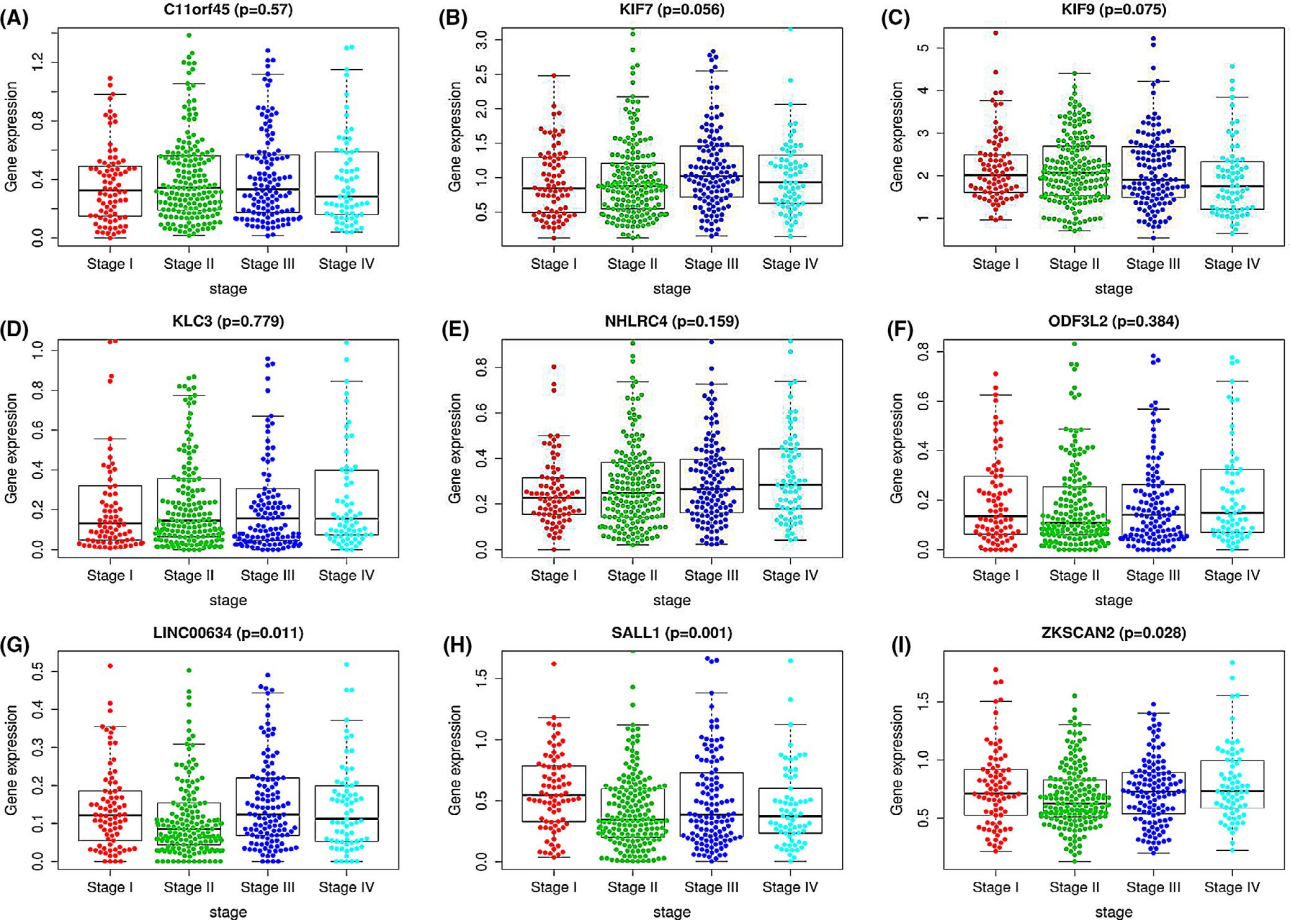
(A–I) The expression levels of the nine genes in CRC patients across different TNM stages

To assess whether the 9‐gene signature can be regarded as an independent prognostic factor for CRC patients’ OS predicting, multivariate Cox regression analysis was executed in a stepwise manner on the whole cohort. Covariables included risk score (high vs. low) and clinicopathological factors such as age, sex, and TNM stage. The results showed that stage (HR = 2.442, 95% CI = 1.845‐3.231, *P* = 4.17E‐10) and risk score (high vs. low) (HR = 4.393, 95% CI = 2.314‐8.339, *P* = 6.01E‐06) could be used as independent prognostic factors (Table 3). Furthermore, we also confirmed the expression level of these nine genes in CRC patients with different TNM stages and found that there were no significant relationships between them (Figure 7). These results proved that our 9‐gene signature’ predictive ability was not related to other conventional clinicopathological factors for predicting OS in CRC patients.

**TABLE 3:** Univariate and multivariate Cox regression analyses of OS in the entire dataset

Variables	Univariate analysis			Multivariate analysis		
	HR	95%CI	*p* value	HR	95%CI	*p* value
Age	1.733	1.138–2.641	0.01	2.206	1.432‐3.401	3.36E−04
Gender	1.039	0.643–1.68	0.875			
Stage	2.511	1.904–3.311	7.09E−11	2.442	1.845‐3.231	4.17E−10
T	2.915	1.818–4.675	9.00E−06			
M	5.196	3.192–8.458	3.37E−11			
N	2.205	1.664–2.921	3.64E−08			
Risk (high vs. low)	5.114	2.730–9.581	3.48E−07	4.393	2.314‐8.339	6.01E−06

Abbreviations: CI, confidence interval; HR, hazard ratio; OS, overall survival.

### 3.4 Combination of age, sex, stage, and 9‐gene signature to construct a nomogram for clinical practice

**FIGURE 8:**
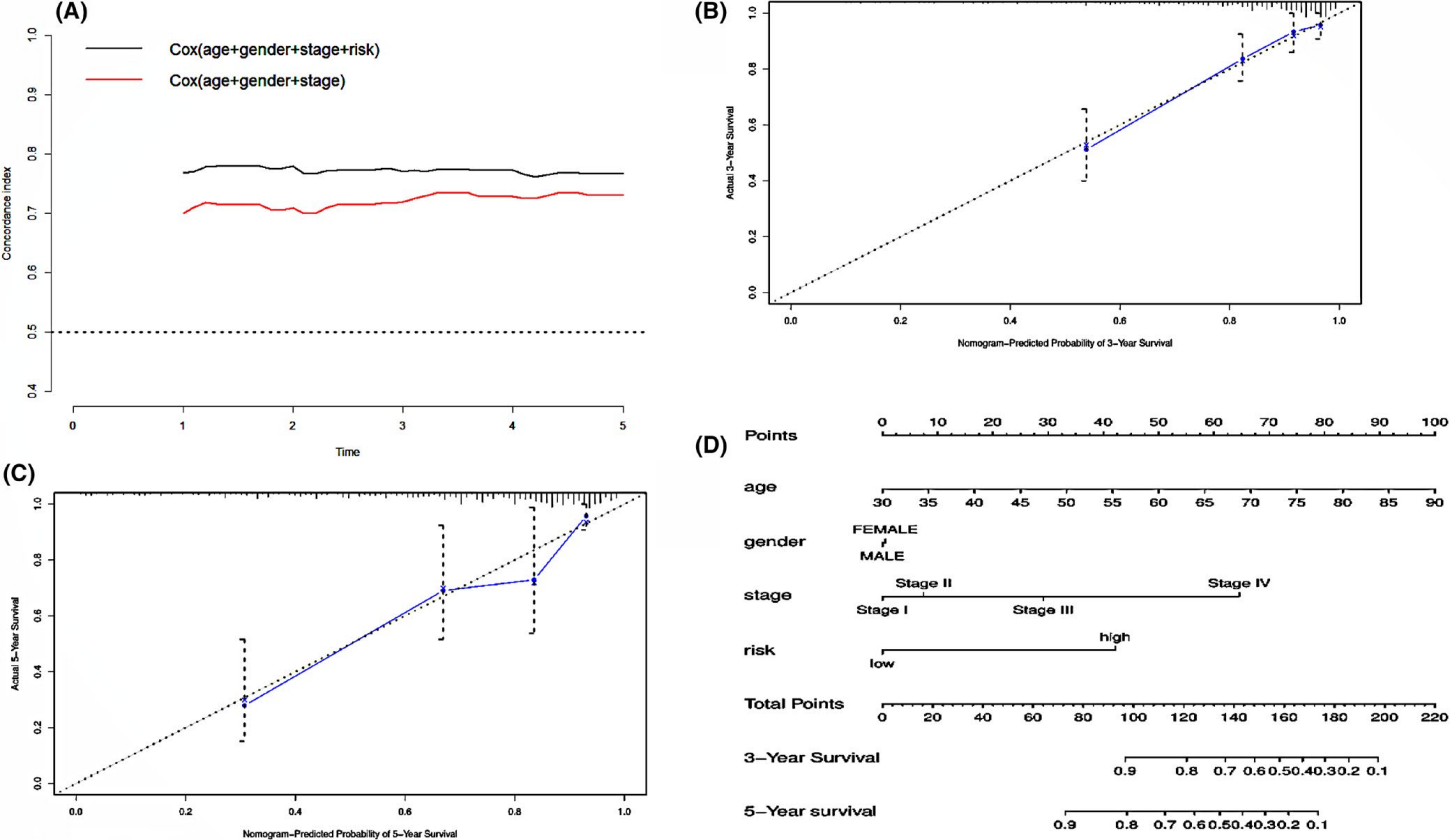
(A) The comparison of C‐index after and before adding the risk grade based on the 9‐gene signature to TNM stage, age, and gender. (B–C) A demonstration of the satisfaction between the predicted and actually 3‐and 5‐year survival in all patients through calibration curves. (D) A nomogram constructed by multi‐cox regression analysis on risk, TNM stage, age, and gender to apply the 9‐gene signature in clinical practice

To apply our findings in clinical practice, we combined the 9‐gene signature with traditional clinicopathological factors such as TNM stage and age to more accurately predict the OS of patients with CRC based on multivariate Cox regression analysis on the entire cohort. To confirm that the accuracy of predicting the OS of CRC patients was improved after adding our 9‐gene signature compared with only those two clinicopathological factors, we calculated the C‐index before and after the addition of our 9‐gene signature. The comparison results elucidated by the C‐index increased from 0.739 (95% CI = 0.676‐0.802) to 0.794 (95% CI = 0.737‐0.851), indicating an obvious increase in C‐index after adding our signature (Figure 8A). Calibration curves showed the degree of consistency between the predicted 3‐and 5‐year survival and the actual survival in all patients (Figure 8B–C). Finally, a nomogram was developed to score each CRC patient based on the factors in the graph (Figure 8D).

### 3.5 Co‐expression relationship between all survival‐related genes and the nine genes in our signature based on WGCNA

**FIGURE 9:**
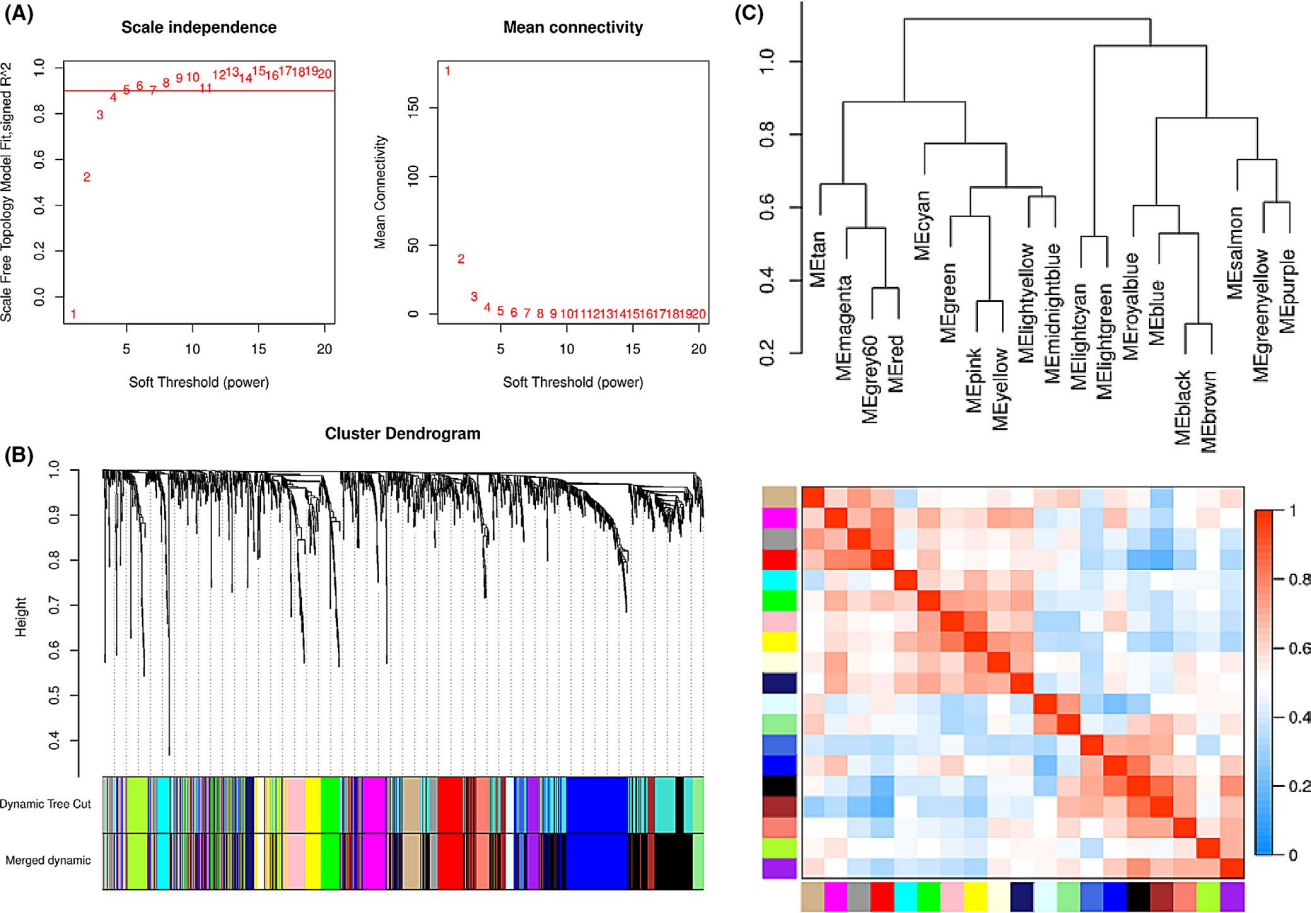
WGCNA was used to identify the co‐expression of survival‐related genes with these nine genes. (A) The soft threshold of a scale‐free topology model. (B–C) The hierarchical clustering tree and its 19 corresponding gene modules

To explore the possible co‐expression relationship between the genes in our signature and other survival‐related genes, we performed WGCNA without clinical traits. With a scale‐free network and topological overlaps, by selecting 5 as the soft threshold and merging similar modules, we generated a hierarchical clustering tree, and 19 corresponding gene modules were identified (Figure 9A,B), in which the branches of the tree on behalf of different gene modules. The non‐co‐expressed genes were disposed of in the “gray” module, which was not further analyzed. The relationships of the 19 modules were analyzed and are shown in Figure 9C. The target gene and its co‐expressed genes were identified from each module and visualized as networks in Cytoscape (Figure [Link], [Link], [Link], [Link], [Link], [Link]). Although the weight values between the genes are relatively low, to some extent, they may still provide clues for fundamental experimental exploration in the future.

## 4 DISCUSSION

CRC is a disease with high intercellular heterogeneity,^11^ which makes traditional clinical predictors such as age, sex, and tumor stage insufficient to accurately predict the survival time of CRC patients.^12^ With the development of genome‐wide sequencing technology, the changes in the genome in CRC are gradually being elucidated by scientists, which also facilitate discovering a series of prognostic and predictive signatures,^13^, ^14^, ^15^ making it possible to make targeted personalized treatment decisions. In this study, by transcriptome expression data from CRC patients, a robust 9‐gene prognostic signature was developed and verified based on the batch screening of genes related to OS in CRC patients.

Of the nine genes (KLC3, LINC00634, NHLRC4, C11orf45, ZKSCAN2, KIF9, KIF7, SALL1, and ODF3L2) in our signature, researchers have proven that SALL1 can act as a tumor suppressor by recruiting NuRD to induce tumor cell senescence in breast cancer during which it is controlled by the MAPK and mTOR signaling pathways.^16^ KLC3 is the only known kinesin light chain (KLC) expressed in postmeiotic male germ cells and researchers have found that KLC3 could play a microtubule‐independent role during the formation of sperm tails.^17^ However, to our knowledge, the role of KLC3 in cancer has not been studied. L. Gomez et al proved that C11orf45 lies within the first intron of KCNJ5 which is associated with Tourette syndrome and attention‐deficit/hyperactivity disorder.^18^ Unfortunately, the function of C11orf45 in the development of tumors has not been studied. Gli2, the major transcriptional activator of Hh signaling, whose localization and activity can be promoted by KIF7, was reported to function in skin development and tumor suppression.^19^ It has been mentioned that KIF9 may be related to the occurrence of breast cancer.^20^ For the other genes in our signature, after searching, we found that they have yet to be studied by scientists. In our research, we found a distinction in the expression levels of these nine genes except for KIF7 between tumor and normal tissues (Figure 5A), indicating that there is great value in exploration.

The 9‐gene signature can classify CRC patients into high‐and low‐risk groups with a significant difference in survival time. It is worth noting that when we tried to prove the reliability of this 9‐gene signature, the results of ROC analysis proved that the AUC values in both the training dataset and test dataset were greater than 0.80 and in the validation dataset it was 0.651, indicating that the nine‐gene combination can be regarded as a reliable and efficient prognostic indicator for CRC patients. Further comparison in the validation dataset showed that the 9‐gene signature was better than Zhou's 5‐gene signature and had an equivalent efficiency as the TNM stage in predicting CRC patients’ 5 years’ OS time verified by AUC values. It should be mentioned that the validation dataset is a collection of drug‐resistant stage II and stage III CRC patients, which suggest that our signature may also benefits drug‐resistant CRC patients with a specific treatment based on the risk score calculated on those nine genes. What is more, the multivariate Cox regression analysis showed that it was independent of other clinical factors, such as age, sex, and stage. TNM stage is the current gold standard for evaluating the prognosis of CRC patients.^3^, ^21^ It is commendable that when we combined the 9‐gene signature with TNM stage and age to evaluate the survival time of patients with CRC, the C‐index increased from 0.739 to 0.794, and the comparison results in Figure 8A show a significantly increase in C‐index after adding the signature, indicating that the 9‐gene signature can be utilized not only as an independent prognostic factor but also as a good supplement to traditional clinicopathological factors to more accurately evaluate the survival of CRC patients.

In conclusion, the 9‐gene signature screened out based on batch survival analysis can be used not only as an independent prognostic index for CRC patients but also as a favorable supplement to traditional clinicopathological factors for clinicians to more accurately and effectively evaluate the survival of CRC patients.

## COMPETING INTERESTS

The authors declare that they have no competing interests.

## Supporting information

Figure S1

Figure S2

Figure S3

Figure S4

Figure S5

Figure S6

Supplementary Material

## Data Availability

The data that support the findings of this study are available from the corresponding author upon reasonable request.
