# Supplementary figures and images for: Improving the overall survival prognosis prediction accuracy: A 9‐gene signature in CRC patients
Source: Cancer Med. 2021 Aug 4;10(17):5998–6009. doi: 10.1002/cam4.4104 (PMC8419765; doi:10.1002/cam4.4104)

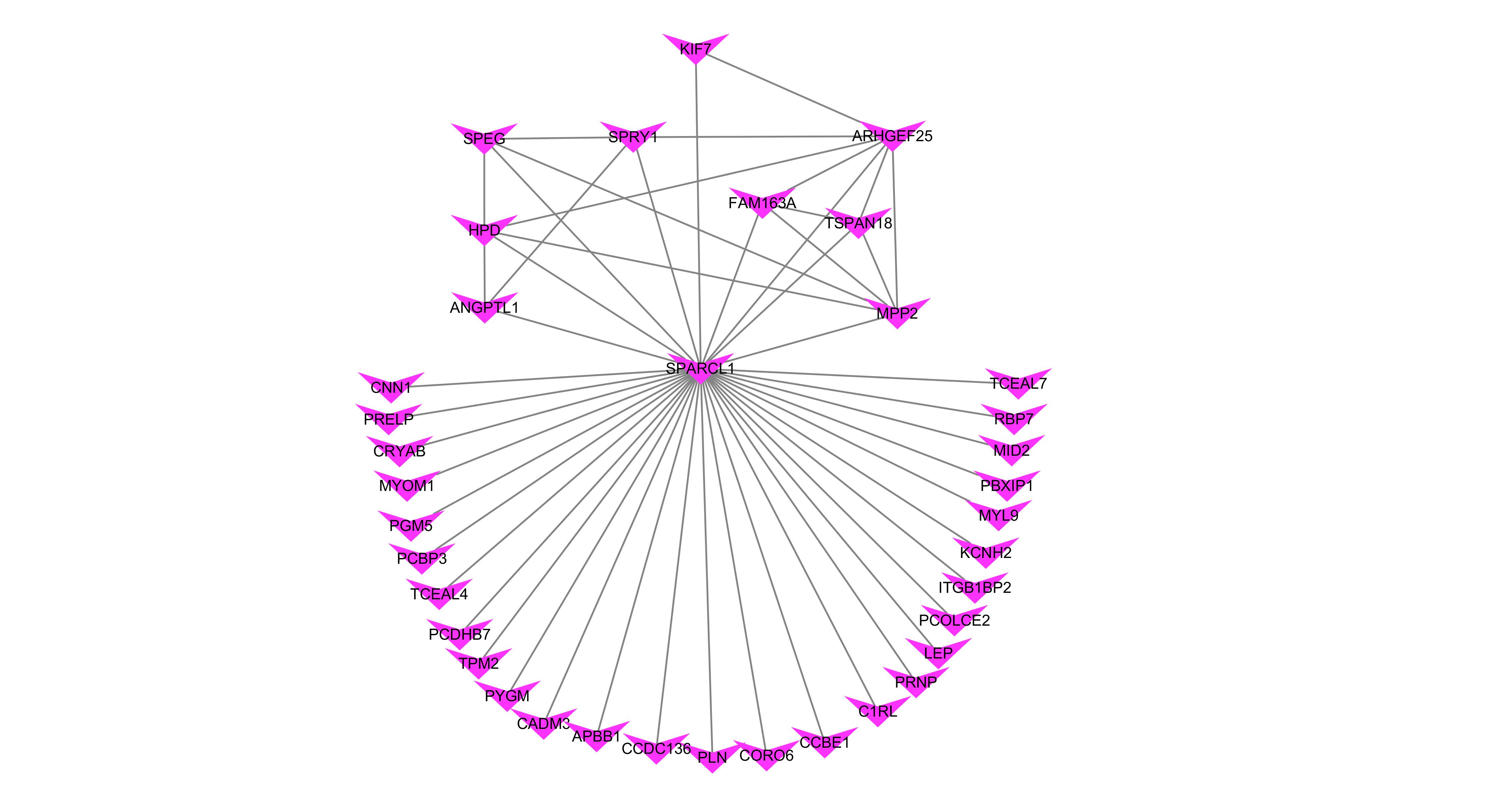

Supplement: Supplementary file 1 — Figure S1 [file CAM4-10-5998-s006.jpeg]

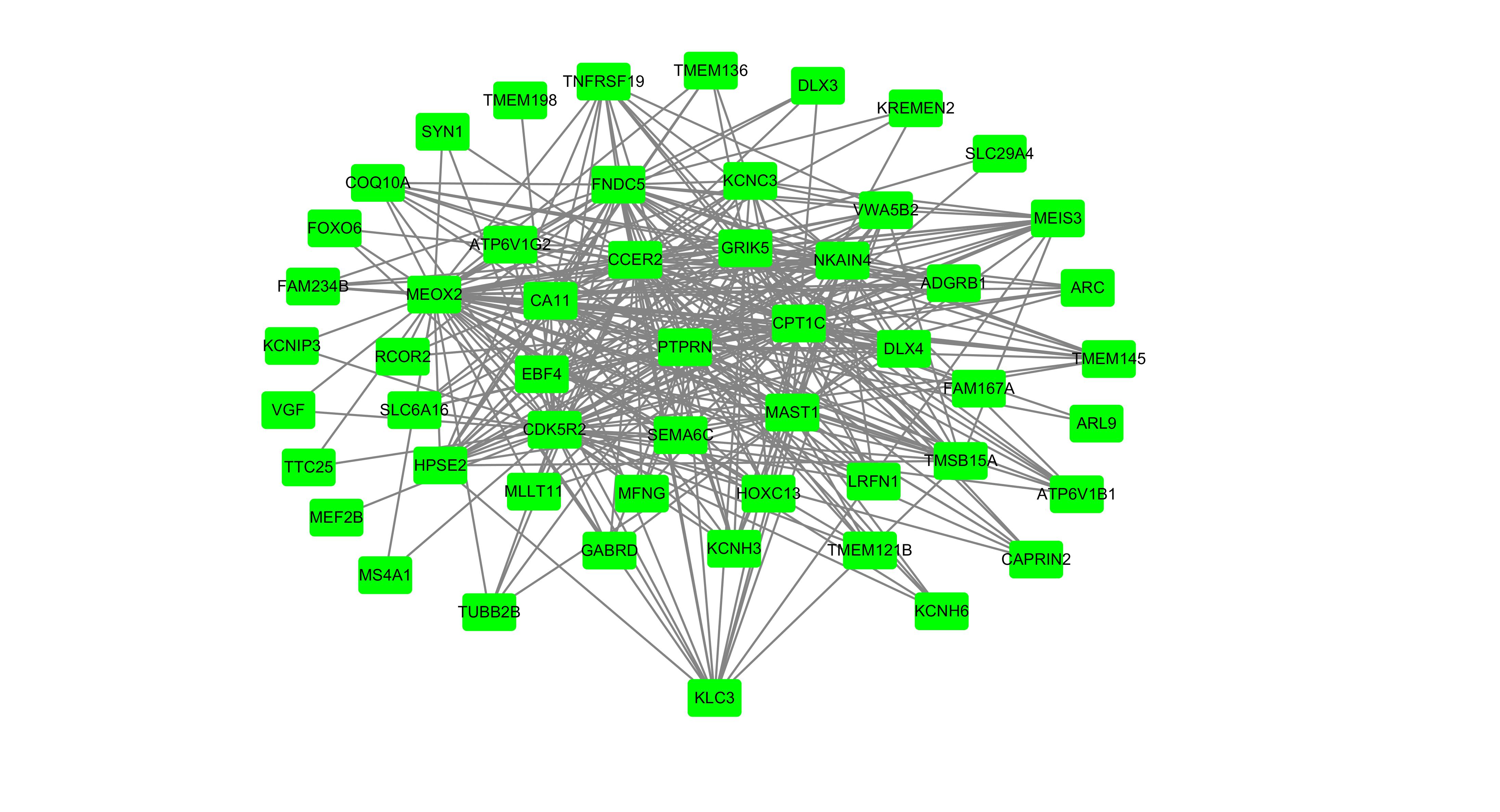

Supplement: Supplementary file 2 — Figure S2 [file CAM4-10-5998-s004.jpeg]

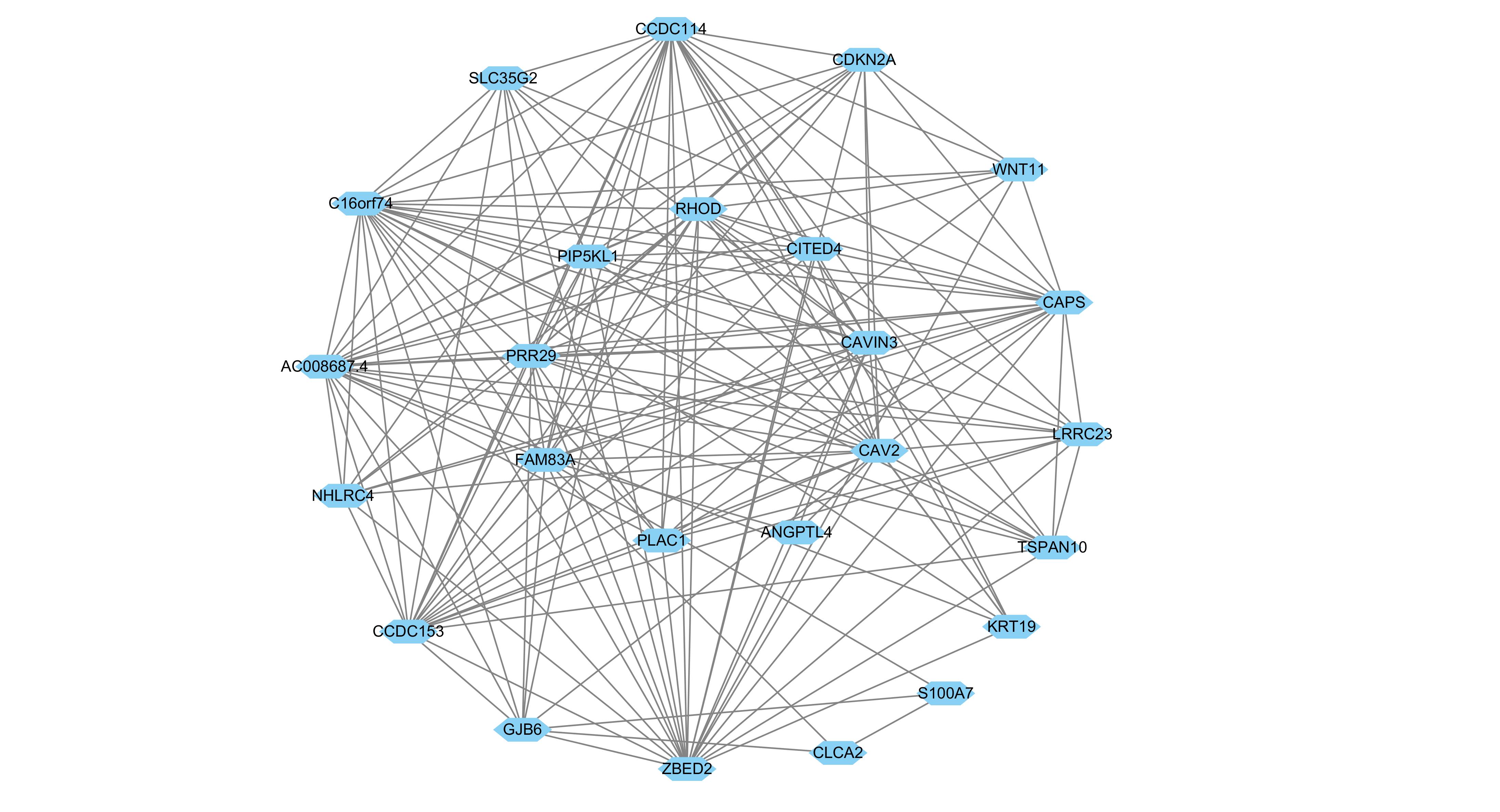

Supplement: Supplementary file 3 — Figure S3 [file CAM4-10-5998-s002.jpeg]

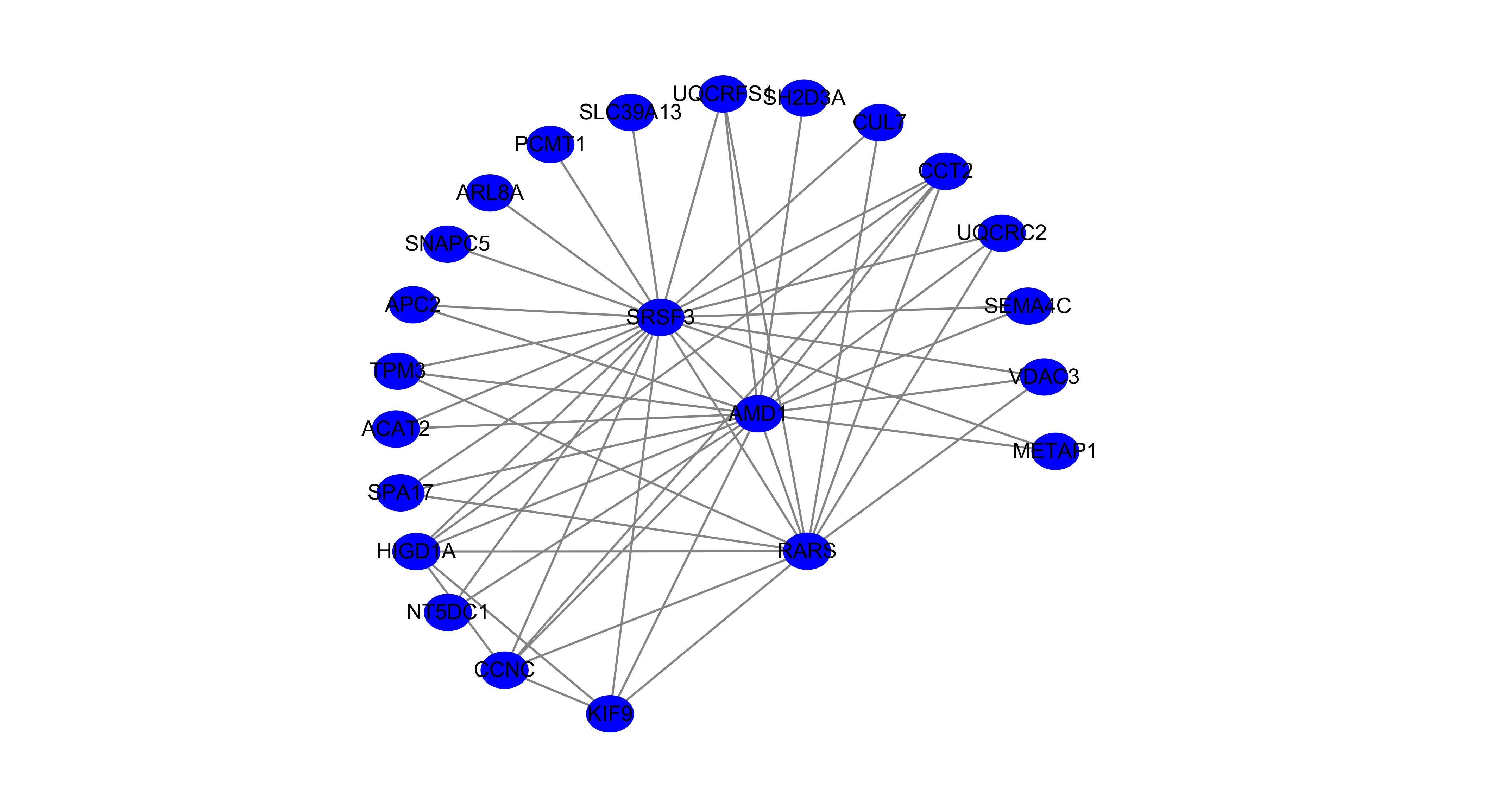

Supplement: Supplementary file 4 — Figure S4 [file CAM4-10-5998-s001.jpeg]

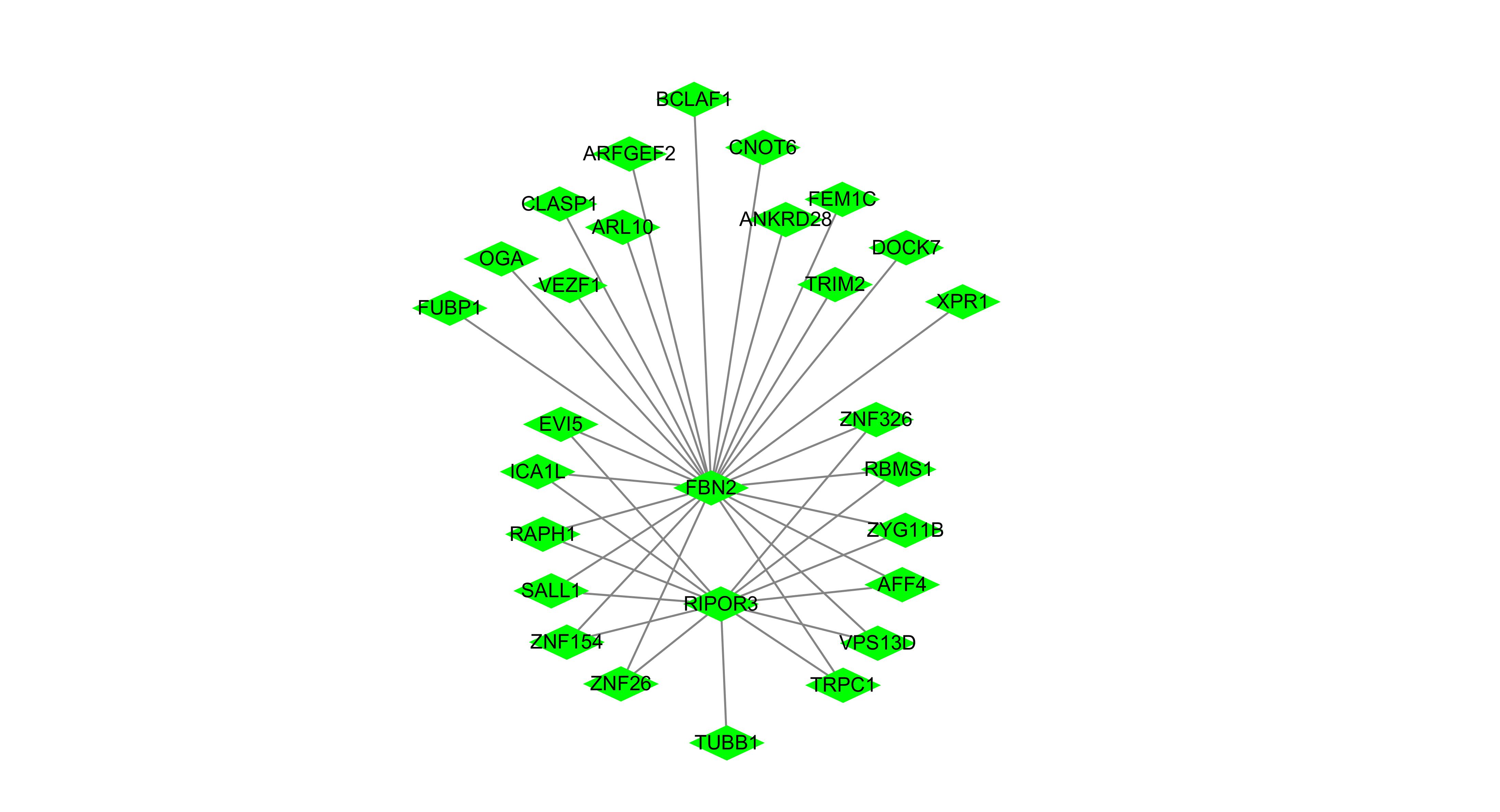

Supplement: Supplementary file 5 — Figure S5 [file CAM4-10-5998-s003.jpeg]

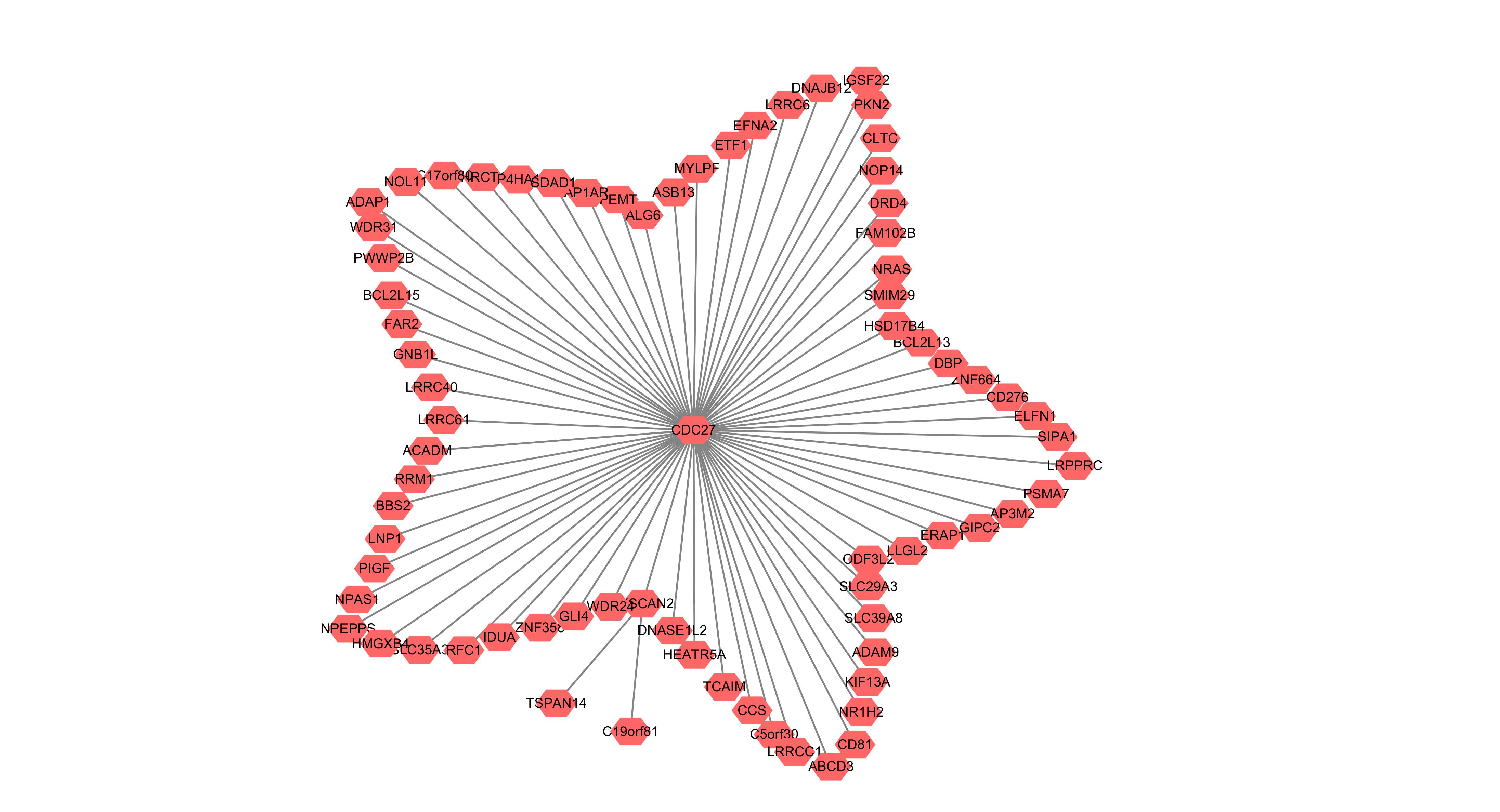

Supplement: Supplementary file 6 — Figure S6 [file CAM4-10-5998-s005.jpeg]
