# Supplementary material for: Improving the overall survival prognosis prediction accuracy: A 9‐gene signature in CRC patients
Source: Cancer Med. 2021 Aug 4;10(17):5998–6009. doi: 10.1002/cam4.4104 (PMC8419765; doi:10.1002/cam4.4104)
Supplement: Supplementary file 7 — Supplementary Material [file CAM4-10-5998-s007.docx]

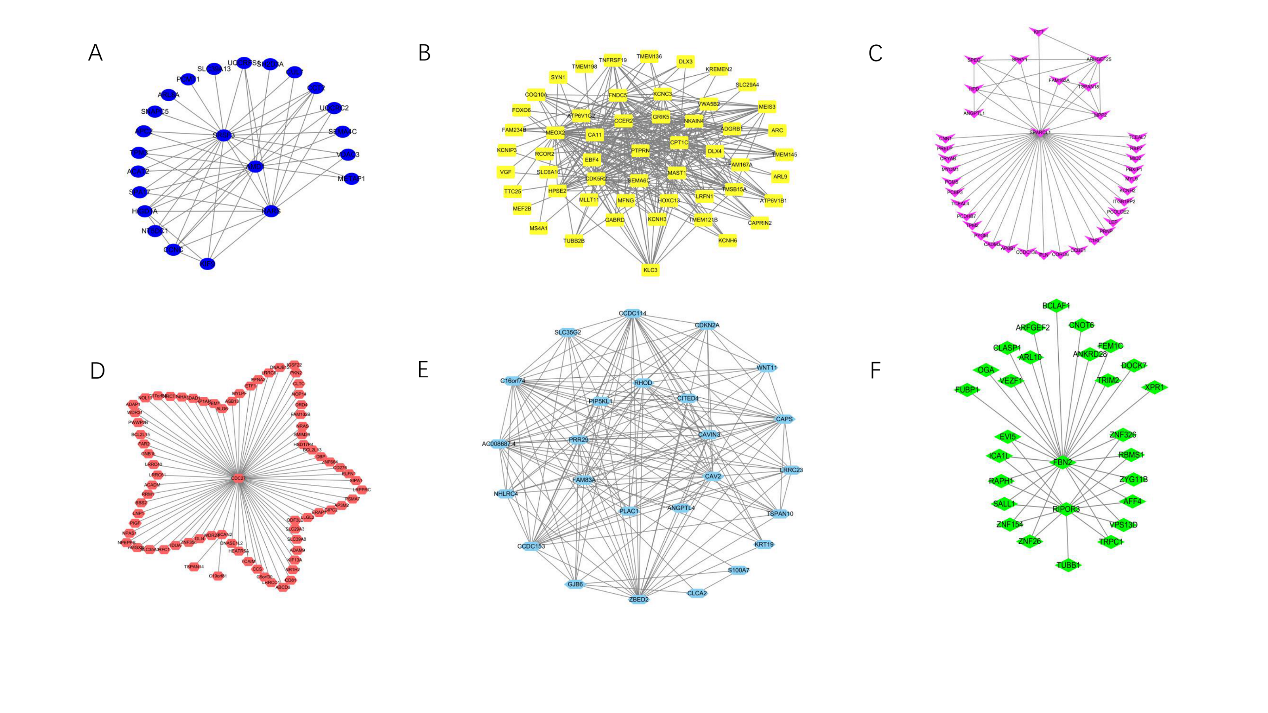


Figure S1-S6. The coexpression networks between the nine genes in our signature and the survival-related genes.
